# Can Plan Recommendations Improve the Coverage Decisions of Vulnerable Populations in Health Insurance Marketplaces?

**DOI:** 10.1371/journal.pone.0151095

**Published:** 2016-03-30

**Authors:** Andrew J. Barnes, Yaniv Hanoch, Thomas Rice

**Affiliations:** 1 Department of Health Behavior and Policy, Virginia Commonwealth University School of Medicine, Richmond, Virginia, United States of America; 2 School of Psychology, Plymouth University, Plymouth, United Kingdom; 3 Department of Health Policy and Management, Fielding School of Public Health, University of California Los Angeles, California, United States of America; Technion Israel Institute of Technology, ISRAEL

## Abstract

**Objective:**

The Affordable Care Act’s marketplaces present an important opportunity for expanding coverage but consumers face enormous challenges in navigating through enrollment and re-enrollment. We tested the effectiveness of a behaviorally informed policy tool—plan recommendations—in improving marketplace decisions.

**Study Setting:**

Data were gathered from a community sample of 656 lower-income, minority, rural residents of Virginia.

**Study Design:**

We conducted an incentive-compatible, computer-based experiment using a hypothetical marketplace like the one consumers face in the federally-facilitated marketplaces, and examined their decision quality. Participants were randomly assigned to a control condition or three types of plan recommendations: social normative, physician, and government. For participants randomized to a plan recommendation condition, the plan that maximized expected earnings, and minimized total expected annual health care costs, was recommended.

**Data Collection:**

Primary data were gathered using an online choice experiment and questionnaire.

**Principal Findings:**

Plan recommendations resulted in a 21 percentage point increase in the probability of choosing the earnings maximizing plan, after controlling for participant characteristics. Two conditions, government or providers recommending the lowest cost plan, resulted in plan choices that lowered annual costs compared to marketplaces where no recommendations were made.

**Conclusions:**

As millions of adults grapple with choosing plans in marketplaces and whether to switch plans during open enrollment, it is time to consider marketplace redesigns and leverage insights from the behavioral sciences to facilitate consumers’ decisions.

## Introduction

The Affordable Care Act (ACA) represents the most sweeping and significant change to the United States health care system since the establishment of Medicare in the 1960’s. The ACA mandates health care coverage for millions of Americans, many purchasing private insurance for the first time, and in the newly developed health insurance marketplaces. During the 2015 open enrollment period, 11.7 million Americans selected a marketplace plan or were automatically re-enrolled, the vast majority of whom received federal premium subsidies.[1] While subsidized insurance with coverage for essential health benefits presents an important opportunity, consumers face enormous challenges in navigating the enrollment and re-enrollment processes. The stakes for users of these marketplaces are high. As demonstrated during the first open enrollment period, signing up for coverage can be daunting.[2] Evidence on how well people are choosing health insurance in the new marketplaces environment is just emerging.

Earlier research examined the quality of insurance choices of low-income, rural individuals with those of an Internet-savvy sample, finding that both groups had difficulty choosing insurance in a hypothetical marketplace that mimicked the federally-facilitated marketplaces, largely because they oftentimes did not choose plans that aligned with their stated preferences (choice consistency), incorrectly answered factual questions about health insurance choices (health insurance comprehension), and could not calculate simple probabilities (numeracy).[3] If people who lack insurance comprehension and numeracy make poorer choices, as we found, it raises the question of whether plan recommendations might result in more effective decision-making. If it does, our results can provide policymakers’ insights into what changes can be made to the marketplace to improve consumers’ decisions. Here we focus on a sample of lower-income, rural, largely low-income Virginians and test each of three types of plan recommendations designed to improve decision-making.

### Previous Research and Hypotheses

Previous experience and data from Massachusetts suggest that 40% of insurance exchange enrollees found health insurance information difficult to comprehend and nearly 20% would like to reduce the number of plan choices.[4] In Medicare Part D, beneficiaries often spend more money than they need to. It has been estimated that, on average, Part D enrollees spend $350-$400 more annually than if they chose the cheapest plan available in their area.[5,6]

Earlier studies have also found numeracy and health insurance comprehension to be critical skills in choosing a health insurance plan that offers consumers adequate risk protection given their expected health care needs [3,7–9]. The relationship between these decision-making skills and the quality of coverage choices was consistent across a spectrum of uninsured individuals differing in age, income, and education. Among individuals likely to enroll in a marketplace plan, fewer than 40% are somewhat or very confident they understand basic insurance terminology.[10] Research shows that even consumers who already possess health insurance often lack basic understanding of and knowledge about their coverage.[11]

Moreover, re-enrollment provides a different set of challenges. During the second annual enrollment period, about half of consumers who chose a plan in 2014 on healthcare.gov and maintained marketplace coverage in 2015 actively renewed their coverage (53%, 2.21 million).[12] The remaining 47% did not actively choose a plan and were automatically re-enrolled in their current plan. In regard to plan switching, in California, about 10 percent of marketplace enrollees switched plans between 2014 and 2015,[13] similar to research on the Medicare prescription drug (Part D) market where 90 percent of beneficiaries stick to their current drug plan during open enrollment.[14,15]. Individuals may stick to their current plan because, among other things, they find it difficult to navigate multiple choices and the accompanying information.[6]

Automatic re-enrollment, which facilitates keeping the status quo plan, is an important convenience to help ensure continuity of coverage. However, it also can result in much higher premiums for marketplace consumers. This is true for two reasons. First, average premium increases between 2014 and 2015 for a particular plan were substantial.[16] Second, federal subsidies, which are used by over 80 percent of marketplace users, are tied to the “benchmark” Silver plan. Premiums for these benchmark plans declined slightly, on average, in 2015, meaning that subsidies declined as well.[17]

Changes in premiums for a given plan do not necessarily mean that people should switch plans. Other considerations, such as the quality of the provider network and disruptions in the continuity of care that could result from plan switching, also must be considered. Indeed, the Obama administration has emphasized the need for all people who have coverage through the marketplaces to visit the website and carefully consider whether the benefits and premiums of their plan in the new year still best meet their needs.[18]

Taken together, these findings raise concerns about consumers’ ability to navigate through the marketplaces, as well as to compare and choose health insurance plans. Providing information that is necessary–but not so much information that it causes more confusion–is the major challenge facing planners of these decision environments. While the designers of the marketplaces have been cognizant of the need for simplicity, preliminary findings indicate that consumers are facing challenges in making cost-effective decisions even in a very simplified version of them.[19]

Emerging work in behavioral economics has attempted to tackle these difficult issues, suggesting ways to redesign decision environments, called “choice architecture,” to aid consumers in making decisions in general and about health insurance in particular.[20] Researchers have proposed a range of options—using defaults, reducing the number of options, providing cost calculators, and tailoring information—that could help consumers make better marketplace decisions and that could influence policymakers.[21,22] Recent evidence from a hypothetical experiment on the influence of choice architecture in the marketplaces suggests labeling plans as Gold, Silver, or Bronze influenced consumers’ preferences for a Gold vs. a Bronze plan, even when the plan attributes were reversed (i.e., a high premium, low deductible Bronze plan and low premium, high deductible Gold plan).[23] Consumers with lower mathematical skills were most susceptible to these labeling effects. Additionally, consumers chose higher premium, lower deductible plans less often when premiums were presented in monthly vs. weekly increments.[23]

Our research follows this promising line of investigation by testing the effectiveness of recommending plans based on lowest annual expected health care costs, a subtle type of change to the marketplace choice architecture. This study examines marketplace plan recommendations from three different norm groups: similar consumers, physicians, and the government. Such changes to the choice architecture have been implemented in the UK to improve tax collection by sending letters to individuals stating that 9 out of 10 people in their area had paid their taxes.[24] Prior evidence from Medicare Part D suggests physicians and friends are among the chief sources consulted by beneficiaries when deciding which plan to choose.[25–29] In another line of investigation, researchers have shown that the majority of Medicare beneficiaries would like to see an active governmental involvement in reducing the number of choices available.[30] Indeed, active purchaser marketplaces limit plan offerings and, in states like California, can tailor the order of plan offerings so that they are presented on the marketplace website based on estimated total costs that includes enrollees’ self-reported expected health care needs.[31]

In this study, a computer-based experiment is conducted using a hypothetical marketplace that mirrors the one consumers face in the federally-facilitated marketplaces, and examines the quality of the decisions they make in choosing health insurance. Of particular interest is whether recommending plans that minimize total expected annual costs can improve consumers’ choices. Decision quality was measured objectively by using an incentive-compatible choice experiment where participant compensation is aligned with performance. Participants were randomly assigned to a control condition, where no recommendation was made, and three conditions where the earnings maximizing plan was recommended. It was hypothesized that individuals assigned to one of the plan recommendation conditions will be more likely to choose the earnings maximizing plan and will earn more than those in the control condition. Additionally, we expect plan recommendations from providers will be more effective than those from the government or based on social norms. Finally, we hypothesize that individuals with lower numeracy or lower insurance comprehension will benefit more from plan recommendations than those with higher numeracy or insurance comprehension.

## Methods

### Data Source

A community sample of individuals residing in the rural southern and southwestern counties of Virginia was recruited using several media outlets including flyers posted in libraries and clinics, advertisement in local newspapers, and through community recruiters. Adults who self-identified as being 18–64 years old were enrolled and asked to complete an online survey and several insurance decision tasks. In total, 690 participants were recruited during the summer of 2014 for a between subjects design where the four plan recommendation conditions (i.e., no recommendation, social normative recommendation, physician recommendation, government recommendation) varied between. Of those, 656 had complete survey data and were used as the analytic sample. Surveys and decision tasks were conducted on computers at local public libraries and a community outreach and research center.

### Insurance Choice Experiment

The insurance choice experiment was designed to span two hypothetical years (Year 1 and Year 2) where participants read scenarios that informed them of the chance they would become ill in the following year and, if ill, would require health care. The probability of illness and cost of care conditional on illness were known. The probability of becoming ill in Year 2 increased if participants became ill in Year 1. If a participant was ill in Year 1, the cost of illness in Year 2 also increased (S1 Appendix).

In each of the two years, participants decided whether or not to buy an insurance plan (Bronze, Silver, or Gold) for the premium amount listed or face a penalty. In total, participants chose from a set of 7 options: 2 Bronze plans (Plans A and B), 2 Silver plans (Plans C and D), 2 Gold plans (Plans E and F), and no insurance. The plan attributes were taken from healthinsurance.gov offerings for single coverage for a nonsmoker living in rural counties in Southern Virginia. These locations were selected due to their high rates of uninsurance as well as their preponderance of low income and minority residents, all of which are of particular importance to efforts to increase marketplace enrollment among vulnerable populations.[32–34]

Plan information given to participants included annual premiums (or the tax penalty for the “no insurance” choice), annual deductible, annual out of pocket maximum, the cost of health care if participants were healthy or sick excluding the premium or penalty, and the total annual costs including premiums or penalties if the participant became ill or remained healthy in the decision scenario. The prompts and decision tasks for the Year 1 and Year 2 scenarios are included in S2 Appendix.

### Quality of Insurance Decisions

The insurance choice experiment was incentivized so that the best coverage decisions were those that earned participants the most money (i.e., choosing a plan that maximized total expected earnings). For example, in Year 1, when the probability of becoming ill and needing to see a doctor is 33% and the cost of illness is $2,000, Plan A is the earnings maximizing choice (Table 1). In Year 2, when the probability of becoming ill and needing to go to the emergency room and stay in the hospital is 80% and the cost of illness is $20,000, choosing Plan E earned participants the most money.

**Table 1:** Expected health care costs in the insurance choice experiment.

	Plan A	Plan B	Plan C	Plan D	Plan E	Plan F	No Insurance
Deductible	$5,500	$4,500	$2,250	$3,350	$2,000	$2,000	N/A
Out of pocket maximum	$6,350	$6,350	$6,350	$5,500	$3,000	$3,000	N/A
*Year 1*							
Total costs if *healthy* and do not need to see a doctor including annual premiums or tax penalties	$108	$156	$400	$492	$1,148	$1,348	$695
Total costs if *ill* and need to see a doctor including annual premiums or tax penalties	$2,108	$2,156	$2,400	$2,492	$3,148	$3,348	$2,695
Total expected health care costs	**$768**	$816	$1,060	$1,152	$1,808	$2,008	$1,355
Year 2 (if ill in Year 1)*							
Total costs if *healthy* and do not need to go to the ER or hospital including annual premiums or tax penalties	$113	$164	$420	$517	$1,205	$1,415	$730
Total costs if *ill* after your ER visit and hospital stay including annual premiums or tax penalties	$6,463	$6,514	$6,495	$6,017	$4,205	$4,415	$20,730
Total expected health care costs	$5,193	$5,244	$5,280	$4,917	**$3,605**	$3,815	$16,730

Notes: Costs in bold represent the plan choice with the lowest expected total annual costs. The chance of getting ill and needing to see a doctor in Year 1 is 33% and the cost is $2,000 before insurance. If a person is ill in Year 1, the chance of getting ill and needing to go to the ER and stay in the hospital in Year 2 is 80% and the cost is $20,000 before insurance.

*If a person is healthy in Year 1, they maintain a 33% chance of becoming ill and needing to see a doctor.

At the beginning of the study, participants were given 10,000 Monopoly dollars and told that each 100 Monopoly dollars is worth one real dollar. Participants were told their payment for the insurance decision task was the average earnings across the Year 1 and Year 2 choices. After participants made their decision in Year 1, they were informed whether they were randomly assigned as healthy or sick, and the costs of premiums (or penalties) and health care services, if sick, are deducted from their 10,000 Monopoly dollars.

Participants were paid a $5 show-up fee, up to approximately $100 for the choice experiment depending on their insurance decisions and chance, and $5 for completing a survey. The average earnings across the Year 1 and Year 2 choices was 8,448 out of the possible 10,000 Monopoly dollars ($84.48 real dollars, SD $14.60, range $36.53-$98.90). Leveraging this incentive-compatible experimental plan choice framework the quality of insurance decisions was assessed using the following two outcomes: 1) an indicator of whether a participant chose the earnings maximizing plan, and 2) the amount of Monopoly dollars earned.

### Plan Recommendations

In Year 2, participants were given another 10,000 Monopoly dollars and were then randomly assigned to a marketplace where either no plan was recommended, or their physician, other enrollees with similar health care needs, or the government recommended a plan to choose. Only the plan that maximized a participant’s expected earnings in the experiment was recommended, although they were not told that explicitly. If participants were healthy in Year 1, a low premium, high deductible plan was recommended in Year 2 (i.e., a Bronze plan). Conversely, if participants were ill in Year 1, a high premium, low deductible plan was recommended in Year 2 (i.e., a Gold plan). Participants were given one of the following prompts: (i) 90% of people with their risk of illness choose the Bronze (Gold) plan; or (ii) their doctor recommends people with their risk of illness choose the Bronze (Gold) plan; or (iii) the government recommends that people with their risk of illness choose the Bronze (Gold) plan. Roughly one-quarter of the sample (24.0%) received no recommendation, while 23.7% received the provider recommendation condition, 22.4% received the social normative condition, and 29.9% received the government recommendation condition. This unequal distribution across conditions occurred because the randomization software in Qualtrics, the survey software, did not evenly allocate individuals across the experimental conditions. The software tended to assign participants to “government plan recommendation” more frequently than other conditions. Nonetheless, the results are not sensitive to randomly dropping observations in the government recommendation condition to create equal groups across all experimental and control conditions.

### Covariates of Interest

Covariate information was collected in following six sections of the survey collected after the choice experiment: 1) demographics, 2) health status,[35] 3) health services utilization,[36] 4) numeracy,[37] 5) risk preferences in the health, gambling, and investment domains assessed using the DOSPERT[38] and time preferences,[39] and 6) actual consumer experiences during the 2013–2014 health insurance marketplace open enrollment period. The study was approved by the Virginia Commonwealth University Institutional Review Board (HM#14789).

### Statistical Analyses

Unadjusted associations between probability of choosing the earnings maximizing plan and plan recommendation conditions were tested using Chi-Square tests, and bivariate associations between participant earnings and the plan recommendation conditions were tested using Student’s t-tests. Generalized estimating equations (GEE) with robust standard errors assuming a binomial distribution of the error terms and a logit link were fit to estimate the adjusted association between the likelihood of choosing the earnings maximizing plan and covariates, with adjusted associations reported as marginal effects. GEE models with robust standard errors assuming normal distribution of the error terms were used to estimate adjusting associations between earnings and covariates. Wald tests were used to determine whether the coefficients of the individual plan recommendation conditions were different from each other. An alpha level of 0.05 was used in all analyses.

## Results

### Sample Characteristics

Of the 656 participants with complete data, the average participant was 40.2 years old (SD 13.5) and most participants were female (67.7%; Table 2). The sample was predominantly African American (78.0%) and the majority had completed at least some college (53.4%). Just under half (49.1%) had never been married. Most participants were healthy with 77.9% indicating their self-rated health status was good, very good, or excellent, and fewer than half (41.9%) reported having any chronic disease or more than one emergency department (ED) visit or any inpatient stays in the past year (37.0%). On average, participants scored near the lower end of the three-item numeracy scale (mean 0.54, SD 0.80) as well as in the gambling and health risk domains, suggesting they tended to be risk averse. Regarding health insurance comprehension and experiences, the average participant correctly answered two of the four health insurance comprehension questions (mean 1.99, SD 1.10). About one-quarter (26.7%) of participants were uninsured and 11.7% had individual private insurance. Finally, 26.5% of participants reported shopping in the marketplace during the 2013–2014 open enrollment season.

**Table 2:** Summary statistics (N = 656 participants).

Variable		Mean or Percent (Std. Dev.)
*Insurance decision quality*	Probability chose Year 1 earnings maximizing plan	41.6%
	Year 1 earnings (Monopoly dollars)*	8,852 (1,054)
	Probability chose Year 2 earnings maximizing plan	48.6%
	Year 2 earnings	7,818 (2,186)
	Average earnings, Years 1 and 2	8,448 (1,460)
*Experimental conditions*	No plan recommendation	24.0%
	Earnings maximizing plan recommended	76.0%
	Doctor recommendation	23.7%
	Social normative	22.4%
	Government recommendation	29.9%
*Demographics*	Age	40.2 (13.5)
	Female	67.7%
	White	18.9%
	African American	78.0%
	Other race	3.0%
	Less than high school	6.9%
	High school or GED	39.8%
	Some college	38.0%
	Bachelors or higher	15.4%
	Married	25.5%
	Widowed or divorced	25.4%
	Never married	49.1%
	Monthly household income quartile	
	$720 or less	24.5%
	$721-$1,200	26.7%
	$1,201–2,499	22.2%
	$2,500 or more	26.5%
*Health status and health care*	Good, very good, excellent health	77.9%
*utilization*	Any Chronic Disease	41.9%
	Current Smoker	31.7%
	More than 1 ED visits and/or any inpatient stay	37.0%
*Numeracy*, *risk and time*	Numeracy (Range 0–3)	0.54 (0.80)
*preferences*	Impatience (Time preference, Range 0–4)	2.06 (1.43)
	Gambling risk (Range 0–5)	0.48 (1.00)
	Investing risk (Range 0–5)	2.42 (1.56)
	Health risk (Range 0–5)	1.21 (1.09)
*Health insurance comprehension*	Health Plan Insurance Comprehension (Range 0–4)	1.99 (1.10)
*and experiences*	No Insurance	26.7%
	Family plan/employer	31.1%
	Individual	11.7%
	Medicare	9.7%
	Medicaid	20.7%
	Shopped in the marketplace	26.5%

Notes

*Participants earned Monopoly dollars but were paid in real dollars by averaging their Monopoly dollar earnings across both years and dividing by 100.

### Associations of Decision Quality in Year 1 and Participant Characteristics

Less than half (41.6%) of participants chose the earnings maximizing plan in Year 1. Relative to Whites, African American participants were 11 percentage points (95% Confidence Interval (CI) 2, 19) more likely to choose a lower premium plan offering less coverage holding all other covariates constant and at the sample average (i.e. the marginal effect), which in Year 1 was the earnings maximizing plan under expectation (Table 3). Similarly, those with less than a high school education were 13 percentage points (95% CI 1, 26) more likely compared to those with a high school degree. Conversely, those with a bachelors or graduate degree were 10 percentage points less likely to choose the earnings maximizing plan in Year 1 (95% CI -20, -1).

**Table 3:** Adjusted Associations of Insurance Decision Quality in Experiment Year 1. (N = 656 participants).

	Percentage Point Change in Probability Chose Year 1 Earnings Maximizing Plan	Year 1 Earnings in Monopoly Dollars
	(95% CI)	(95% CI)
Age	0	-5
	(0, 0)	(-11, 0)
Female	6	92
	(-2, 13)	(-54, 239)
African American	11*	-113
	(2, 19)	(-280, 55)
Other race	18*	-158
	(-1, 38)	(-537, 221)
Less than high school	13*	158
	(1, 26)	(-114, 431)
Some college	0	37
	(-8, 8)	(-110, 184)
Bachelor’s degree or higher	-10*	-91
	(-20, 1)	(-271, 89)
Married	1	61
	(-8, 11)	(-116, 238)
Widowed or divorced	8	-24
	(-1, 17)	(-202, 154)
Monthly household income quartile		
$721-$1,200	-2	105
	(-11, 7)	(-70, 279)
$1,201–2,499	-5	-56
	(-16, 5)	(-252, 140)
$2,500 or more	-10	-84
	(-21, 0)	(-289, 120)
Good, very good, excellent health	8	206*
	(0, 16)	(43, 369)
Any Chronic Disease	-2	68
	(-9, 5)	(-75, 211)
Current Smoker	-3	58
	(-10, 4)	(-81, 196)
More than 1 ED visits and/or any inpatient stay	1	-43
	(-6, 8)	(-180, 93)
Numeracy	4	2
	(-1, 8)	(-77, 82)
Impatience (Time preference)	0	54*
	(-3, 2)	(12, 96)
Gambling risk	2*	-30
	(-2, 5)	(-97, 37)
Investing risk	-3*	23
	(-5, -1)	(-16, 63)
Health risk	-2	24
	(-6, 1)	(-44, 92)
Health Plan Insurance Comprehension	0	-67*
	(-3, 3)	(-127, -7)
No Insurance	2	-82
	(-7, 12)	(-272, 108)
Individual	4	45
	(-7, 12)	(-142, 233)
Medicare	-3	-43
	(-16, 11)	(-285, 199)
Medicaid	3	-60
	(-7, 13)	(-267, 146)
Shopped in the marketplace	3	48
	(-5, 10)	(-92, 188)
Constant	N/A	8,876*
		(8,439, 9,314)

Notes

*p<0.05. Adjusted associations of experimental conditions, covariates and decision quality outcomes were estimated using generalized estimating equations with robust standard errors. Marginal effects are reported for the probability of choosing the earnings maximizing plan holding covariates constant and at the sample average.

### Associations of Decision Quality in Year 2 and Plan Recommendations

#### Unadjusted associations between plan choice, earnings, and plan recommendations

**Fig 1:**
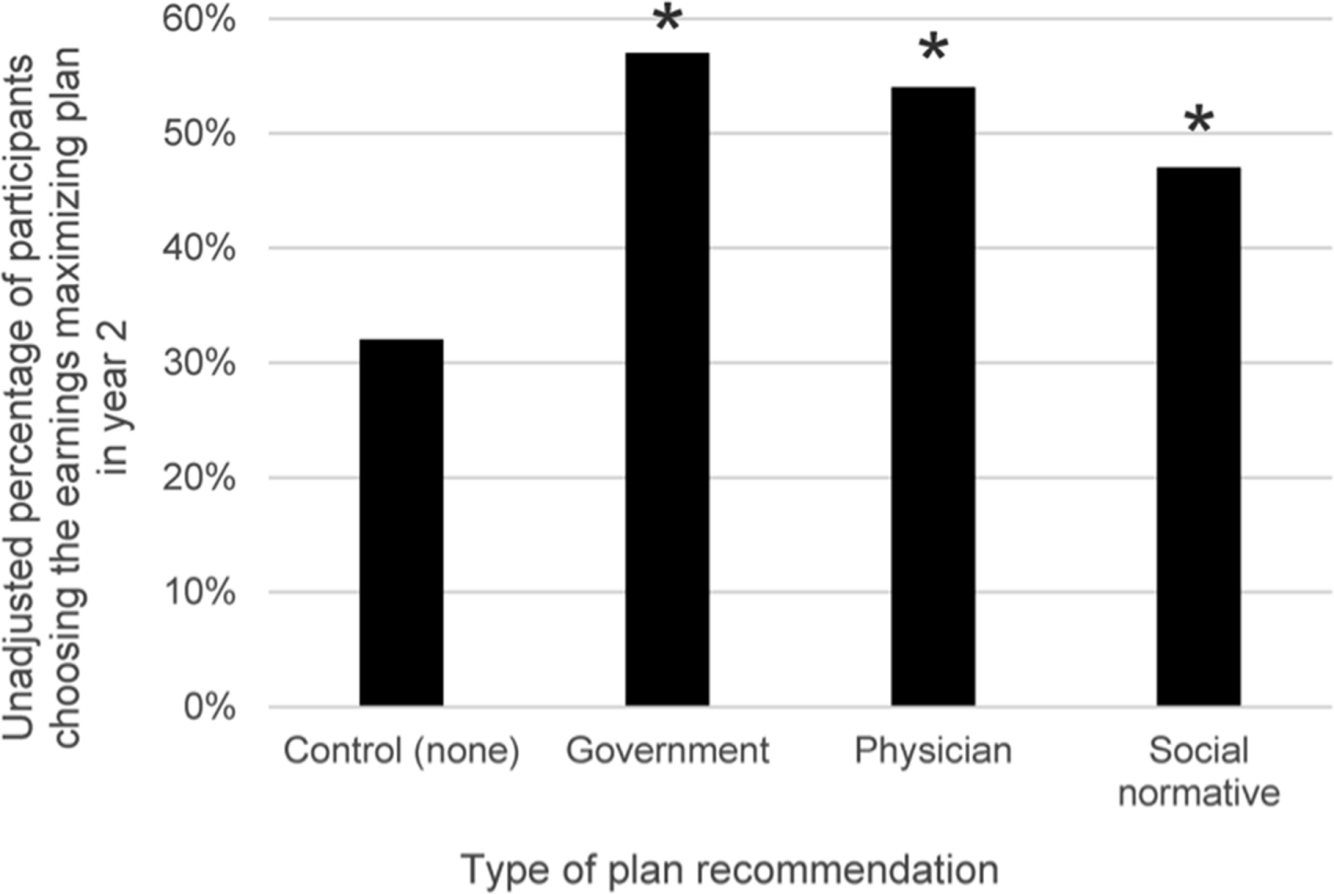
Participants choosing the earnings maximizing plan in Year 2, by experimental condition. Asterisks (*) indicate a statistically significant unadjusted difference compared to the control condition at p<0.05.

In Year 2, before adjustment, 53% of participants chose the earnings maximizing plan when experiencing any of the plan recommendation conditions compared to 32% in the control condition (p<0.05). Specifically, 54% of participants assigned to the physician recommendation, 47% assigned to the social normative recommendation, and 57% assigned to the government recommendation condition chose the earnings maximizing plan (p<0.05, Fig 1).

Participants earned 384 more Monopoly dollars (95% CI 109, 660) when assigned to a choice environment where the plan that maximized expected earnings was recommended compared to participants choosing health insurance without a recommendation, prior to adjustment. Regarding the type of recommendation, participants assigned to the government recommendation condition earned 475 more Monopoly dollars (95% CI 154, 797) than those choosing without any recommendation. Similarly, the participants choosing in the physician recommendation condition earned an additional 378 Monopoly dollars (95% CI 26, 729). There were no earnings differences between participants choosing in the social normative condition and those choosing without any recommendation (256; 95% CI -120, 632). Earnings differentials across plan recommendation conditions averaged across Years 1 and 2 showed a similar pattern.

#### Adjusted associations between choosing the earnings maximizing plan and plan recommendations

After adjustment for demographic characteristics, health status, recent health care utilization history, numeracy, risk and time preference, and health insurance comprehension and experiences, participants assigned to any of the plan recommendation conditions were 21 percentage points (95% CI 15, 28) more likely to chose the earnings maximizing plan than those assigned to the control condition (Table 4). The hypothetical marketplace offering a provider recommendation for a plan resulted in a 23 percentage point (95% CI 14, 31) increase in the probability of choosing the earnings maximizing plan. Participants choosing in a marketplace where 90% of people with their risk of illness recommended a plan were 16 percentage points (95% CI 8, 24) more likely and those randomized to a marketplace where the government recommended a plan were 24 percentage points (95% CI 17, 32) more likely to choose the earnings maximizing plan. In regard to control variables, African Americans were more likely than Whites to choose the earnings maximizing plan (p<0.05) as were participants who were previously married compared to those who were currently married (p<0.05).

**Table 4:** Adjusted Associations of Plan Recommendation Conditions and Choosing the Earnings Maximizing Plan in Year 2 (N = 656 participants).

	Percentage Point Change in Probability Chose Year 2 Earnings Maximizing Plan	
	(95% CI)	
Any recommendation vs. none	21*	---
	(15, 28)	
Doctor recommendation vs. none	---	23*
		(14, 31)
Social norm recommendation vs. none	---	16*
		(8, 24)
Government recommendation vs. none	---	24*
		(17, 32)
Age	0	-0.27
	(0, 0)	(-1, 0)
Female	6	7
	(-1, 13)	(0, 14)
African American	12*	12*
	(4, 20)	(4, 20)
Other race	17	17
	(-3, 37)	(-2, 37)
Less than high school	6	5
	(-7, 19)	(-7, 18)
Some college	-1	-1
	(-8, 7)	(8, 6)
Bachelor’s degree or higher	-7	-6
	(-17, 3%)	(-16, 4)
Married	5	6
	(-3, 14)	(-2, 15)
Widowed or divorced	9*	10*
	(1, 18)	(2, 19)
Monthly household income quartile		
$721-$1,200	3	2
	(-6, 11)	(-7, 10)
$1,201–2,499	0	1
	(-9, 10)	(-9, 9)
$2,500 or more	-7	-8
	(-17, 3)	(-17, 2)
Good, very good, excellent health	-4	-4
	(-12, 4)	(-12, 4)
Any Chronic Disease	-3	-3
	(-9, 4)	(-10, 4)
Current Smoker	-1	-1
	(-8, 7)	(-8, 7)
More than 1 ED visits and/or any inpatient stay	6	6
	(-1, 12)	(0, 13)
Numeracy	2	1
	(-2, 6)	(-3, 5)
Impatience (Time preference)	-1	-1
	(-3, 1)	(-3, 1)
Gambling risk	0	0
	(-3, 4)	(-3, 4)
Investing risk	-2	-2
	(-4, 0)	(-3, 0)
Health risk	2	2
	(-2, 5)	(-2, 5)
Health Plan Insurance Comprehension	2	1
	(-1, 6)	(-2, 4)
No Insurance	0	0
	(-9, 9)	(-9, 10)
Individual	-4	-4
	(-14, 7)	(-14, 7)
Medicare	-9	-9
	(-20, 2)	(-20, 2)
Medicaid	1	0
	(-9, 10)	(-10, 9)
Shopped in the marketplace	1	1
	(-6, 8)	(-6, 8)
Constant	N/A	N/A

Notes

*p<0.05. Adjusted associations of experimental conditions, covariates and decision quality outcomes were estimated using generalized estimating equations with robust standard errors. Marginal effects are reported for the probability of choosing the earnings maximizing plan holding covariates constant and at the sample average.

#### Adjusted associations between earnings and plan recommendations

The unadjusted associations between Year 2 earnings and plan recommendations were smaller in magnitude but otherwise similar after adjustment (Table 5). Specifically, participants assigned to a marketplace where plans were recommended earned 311 (95% CI 39, 584) more Monopoly dollars in Year 2 than participants who were assigned to the marketplace without a recommendation.

**Table 5:** Adjusted Associations of Plan Recommendation Conditions and Earnings (N = 656 participants).

	Year 2 Earnings	Year 2 Earnings	Average Earnings Across Years 1 & 2	Average Earnings Across Years 1 & 2
	(95% CI)	(95% CI)	(95% CI)	(95% CI)
Any recommendation vs. none	311*	---	217*	---
	(39, 584)		(28, 405)	
Doctor recommendation vs. none	---	360*	---	252*
		(23, 698)		(18, 489)
Social norm recommendation vs. none	---	160	---	139
		(-185, 504)		(-99, 378)
Government recommendation vs. none	---	391*	---	249*
		(77, 705)		(30, 468)
Age	-8	-9	-7	-7
	(-20, 3)	(-20, 2)	(-15, 1)	(-15, 1)
Female	114	128	108	112
	(-153, 380)	(-142, 397)	(-82, 298)	(-79, 303)
African American	-396*	-403*	-251*	-253*
	(-690, -101)	(-698, -108)	(-465, -38)	(-467, -40)
Other race	-341	-338	-233	-233
	(-923, 242)	(-925, 249)	(-673, 207)	(-677, 212)
Less than high school	58	51	108	106
	(-451, 567)	(-457, 558)	(-255, 472)	(-256, 467)
Some college	-26	-37	1	-4
	(-302, 249)	(-314, 240)	(-192, 195)	(-198, 191)
Bachelor’s degree or higher	-28	-25	-64	-63
	(-393, 337)	(-393, 342)	(-312, 184)	(-313, 186)
Married	264	269	156	162
	(-60, 588)	(-58, 596)	(-72, 384)	(-69, 392)
Widowed or divorced	-43	-34	-38	-29
	(-376, 291)	(-367, 299)	(-271, 195)	(-262, 205)
Monthly household income quartile				
$721-$1,200	53	48	74	74
	(-273, 379)	(-277, 374)	(-153, 301)	(-152, 301)
$1,201–2,499	-149	-147	-100	-101
	(-511, 213)	(-507, 213)	(-354, 153)	(-353, 153)
$2,500 or more	-364	-358	-220	-219
	(-752, 24)	(-745, 29)	(-491, 51)	(-490, 52)
Good, very good, excellent health	443*	448*	321*	325*
	(136, 750)	(142, 754)	(107, 535)	(111, 538)
Any Chronic Disease	277*	284*	171	175
	(8, 545)	(15, 553)	(-17, 360)	(-14, 364)
Current Smoker	-23	-31	17	11
	(-284, 238)	(-292, 230)	(-168, 201)	(-172, 196)
More than 1 ED visits and/or any inpatient stay	92	81	23	15
	(-163, 347)	(-176, 337)	(-158, 203)	(-166, 196)
Numeracy	-73	-78	-35	-37
	(-224, 78)	(-229, 73)	(-141, 72)	(-144, 69)
Impatience (Time preference)	21	23	38	38
	(-59, 100)	(-56, 102)	(-17, 93)	(-17, 93)
Gambling risk	-95	-93	-62	-62
	(-220, 30)	(-218, 32)	(-150, 26)	(-150, 26)
Investing risk	57	59	40	42
	(-17, 131)	(-14, 133)	(-12, 92)	(-10, 94)
Health risk	25	14	24	18
	(-126, 176)	(-137, 164)	(-78, 126)	(-83, 120)
Health Plan Insurance Comprehension	-1	-8	-33	-37
	(-115, 113)	(-121, 104)	(-114, 47)	(-117, 43)
No Insurance	-92	-78	-87	-81
	(-470, 286)	(-457, 301)	(-353, 178)	(-348, 185)
Individual	12	16	22	29
	(-375, 399)	(-370, 402)	(-238, 282)	(-231, 289)
Medicare	-310	-278	-181	-166
	(-756, 136)	(-724, 167)	(-490, 129)	(-476, 144)
Medicaid	-11	-3	-35	-31
	(-390, 367)	(-383, 377)	(-304, 234)	(-301, 238)
Shopped in the marketplace	50	40	41	38
	(-239, 339)	(-249, 329)	(-157, 239)	(-160, 236)
Constant	7,673*	7,703*	8,224*	8,243*
	(6,827, 8,518)	(6,865, 8,541)	(7,630, 8,817)	(7,653, 8,832)

Notes

*p<0.05. Adjusted associations of experimental conditions, covariates and earnings were estimated using generalized estimating equations with robust standard errors.

Participants choosing in a marketplace that offered a provider recommendation for a health insurance plan that maximized expected earnings received an additional 360 Monopoly dollars (95% CI 23, 698), on average, compared to those choosing without any recommendation. Similarly, participants selecting a plan in a marketplace where the government recommended the earnings-maximizing plan earned 391 (95% CI 77, 705) more Monopoly dollars than those choosing without a recommendation. Earnings did not differ significantly after adjustment between the marketplace and with social normative plan recommendations the marketplace without any plan recommendations (160, 95% CI -185, 504). The adjusted effects of the plan recommendation conditions on earnings averaged across Years 1 and 2 were similar.

With few exceptions, the control variables were not associated with Year 2 or average Years 1 and 2 earnings in the adjusted models. However, African Americans had lower earnings, on average, compared to White participants, while participants in good, very good, or excellent health earned more compared to those in poor or very poor health.

#### Subgroup analyses and sensitivity tests

To test the robustness of the main findings presented above, bivariate and multivariate subgroup analyses were conducted to test for differences in decision quality across those randomly assigned to “sick” and “well” health states after Year 1, as well as test differences across participants who had and had not previously shopped in the health insurance marketplace. In the first subgroup analyses, participants were stratified by whether they were randomly assigned to a “sick” or “well” condition after Year 1. Results from bivariate Chi-square tests provided insight into findings from the main adjusted models presented above that African Americans were simultaneously more likely to choose the earnings maximizing plan and earn less money. For participants assigned to the “well” condition where the low coverage option with the lowest premium (Plan A) is the earnings maximizing plan, Africans Americans were significantly more likely than Whites to select this plan (p<0.05). However, for participants assigned to the “sick” condition, where the earnings maximizing plan is the high coverage option with the lowest premium (Plan E), African Americans and Whites choose Plan E with equal likelihood but African Americans are more likely to choose Plan A (p<0.05), hence incur larger losses.

The main regression models above were also stratified by health condition assignment after Year 1 and the results suggest that health insurance comprehension is a significant positive predictor of Year 2 decision quality among those assigned to the “sick” condition (p<0.05) but not among those assigned to the “well” condition. Also, importantly, plan recommendations significantly improve the probability of choosing the earnings maximizing plan, regardless of health condition assignment (p<0.05 in both stratified models). In regard to prior marketplace experiences, assignment to any plan recommendation condition significantly increased the probability of choosing the earnings maximizing plan for participants with and without prior marketplace experience (p<0.05 each). However, recommendations were not associated with higher earnings among those who reported previously shopping in the marketplace.

The main findings were not sensitive to the distribution of the earnings outcome. Log-transformed earnings models yielded equivalent results in terms of the sign and compactness of the estimates. Also, no significant interactions between the plan recommendation conditions and education, race/ethnicity, self-reported health status, or insurance comprehension were found. Importantly however, the association between plan recommendations and the probability of choosing the earnings maximizing plan was significantly moderated by numeracy (p<0.05) such that those with lower levels of numeracy benefitted more from plan recommendations compared to those with higher levels of numeracy.

## Discussion

The U.S. Department of Health & Human Services reported that roughly half of those enrolled in 2014 actively renewed their plan during the open enrollment period, despite the fact that switching plans has the potential to substantially reduce cost.[12] This study is the first incentive-based experiment, to our knowledge, to investigate the use of plan recommendations, a form of altering the choice architecture, to improve marketplace decisions. As advocated by others,[20,21] the results support the first hypothesis that plan recommendations improve decision-making in the hypothetical experiment. When choosing insurance without a plan recommendation, less than half chose the earnings maximizing plan in Year 1 (42%) or Year 2 (32%). Plan recommendations increased the probability of choosing the plan that minimized total expected annual spending on health by 21 percentage points after adjustment illustrating the potential of introducing similar behaviorally informed policy tools into the Affordable Care Act decision environment to benefit consumers’ choices.

Inspired by earlier evidence on Medicare and investigations conducted by the U.K. government, the data indicate that two out of the three plan recommendation conditions—physician recommendation and government recommendation—resulted in significantly better coverage decisions. In contrast to the second hypothesis, provider and government recommendations appeared to have equivalent effect sizes, although social normative recommendations did appear to have smaller effects in some models. Earlier work on Medicare Part D suggests that the majority of older adults would prefer the government to take a more active role designing insurance markets to improve decision-making.[30] It is also aligned with reports indicating that many older adults viewed their physicians as one of the main sources of information about which Medicare part D plan to choose.[25–29] This evidence highlights the positive value nonelderly consumers place on these sources of information about health care coverage. Regarding the null finding for the association between social normative plan recommendations and decision quality, it is possible that since many participants had difficulty understanding and choosing a plan in the experiment they would be skeptical that “90% of people like them” would have any more information about choosing a plan. There was limited support for the third hypothesis that plan recommendations were more effective for consumers with lower levels of numeracy or health insurance comprehension. Specifically, participants with lower levels of numeracy were more likely to choose an earnings maximizing plan when choosing in one of the plan recommendation conditions compared to those with higher levels of numeracy. However, we found no evidence of a similar moderating effect of health insurance comprehension.

Several important limitations should be considered when evaluating the implications of the study. First, the experiment approach employed cannot capture the complexity of actual consumer decision-making and the markets they choose in. For example, the role of networks and preferences for providers is not addressed in the experimental design tested. Prior work suggests concerns about coverage and access are very important in choosing a plan and that plan choosers, correctly or not, place a high value on the ability to keep their current physician network of other providers.[40] Moreover, the effectiveness of the plan recommendations tested depend on full information on both the probability of becoming ill and needing care and the conditional expected spending. The ability to provide such recommendations in actual insurance choice environments based on this level of detail is limited. Nonetheless, plan choice architecture can incorporate prior spending and health care utilization profiles and expected changes to these historical profiles to predict future utilization and spending and recommend plans that minimize expected total annual costs. Indeed the federally-facilitated marketplace in for the 2015–2016 open enrollment period includes estimated annual costs for consumers based on three broad categories of expected health care utilization (e.g. low, medium, high). Although the hypothetical choice study does not fully reflect the dynamics of real-world choices, the incentive-compatible design used to align payoffs with the quality of insurance decisions certainly mitigates some of the concern about the external validity of the results presented.

Second, the data were gathered from a community sample of participants residing in several rural counties in Southern Virginia and so the results may not generalize to those choosing in marketplaces throughout the U.S. Also, the marketplaces in the experiment were modeled on the federally-facilitated marketplaces and the findings may not extend to state-based marketplaces where states have made more efforts to improve the comparability of plans when shopping. Finally, plan recommendations are a subtle form of changing the choice architecture. The psychological and behavioral economics literature offers a spectrum of different ways in which the choice architecture of marketplaces could have other important influences on consumer enrollment and re-enrollment choices.[21,41]

Importantly, the results suggest that plan recommendations may be beneficial to a wide array of consumers. The simplicity of plan recommendations, like those employed–either one follows them or not—renders them attractive policy options for improving decision-making broadly without limiting consumers’ autonomy of choice over the plans offered in the market. Such evidence is again supported in the Medicare prescription drug market where beneficiaries were more likely to switch plans when sent a letter about which plan offering lowered their expected prescription drug coverage costs.[42] Given the preponderance of consumers who are not actively shopping for marketplace plans, creating simpler and easier means to evaluate plans could have substantial impact on consumers’ annual health care costs, the number of individuals who switch plans, as well as the value of the federal subsidies provided. Additionally, employing similar forms of choice architecture may benefit more advantaged and disadvantaged marketplace consumers. With the marketplaces having overcome troubled rollouts and succeeded in robust re-enrollment periods in the second year, they have entered a third phase, recently referred to as “Healthcare.gov 3.0.”[23] Looking ahead in this new phase, millions of adults will continue grappling with which insurance plan to choose and whether to switch plans each year during open enrollment. Now is the time to consider marketplace redesigns that leverage insights from the behavioral sciences to facilitate and improve consumers’ decisions.

## Supporting Information

S1 Appendix Experimental Design. (DOCX)

S2 Appendix Decision Tasks. (DOCX)
