# Supplementary figures and images for: Can Plan Recommendations Improve the Coverage Decisions of Vulnerable Populations in Health Insurance Marketplaces?
Source: PLoS One. 2016 Mar 30;11(3):e0151095. doi: 10.1371/journal.pone.0151095 (PMC4814125; doi:10.1371/journal.pone.0151095)

**S1 Appendix Experimental Design**


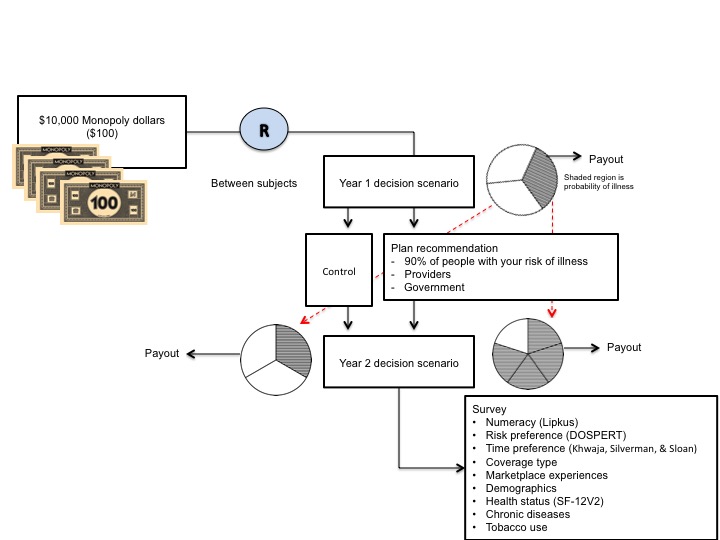

Supplement: S1 Appendix — (DOCX) [file pone.0151095.s001.docx]
