# Supplementary material for: Can Plan Recommendations Improve the Coverage Decisions of Vulnerable Populations in Health Insurance Marketplaces?
Source: PLoS One. 2016 Mar 30;11(3):e0151095. doi: 10.1371/journal.pone.0151095 (PMC4814125; doi:10.1371/journal.pone.0151095)
Supplement: S2 Appendix — (DOCX) [file pone.0151095.s002.docx]

**S2 Appendix Decision Tasks**

**Year 1 Prompt**

You have just received $10,000 Monopoly dollars. In Task 1, you will be given two scenarios where you have a chance of getting sick in the next year. Next, you will be asked whether you want to spend some of your Monopoly dollars to buy health insurance for the year to cover some of your medical costs if you get sick. Both of the scenarios represent a separate year for a total of two years.

Your choices have real financial consequences. Every 100 Monopoly dollars is worth 1 real dollar. Each year in Task 1 you can earn between $0 and $100, you cannot lose money. How much you earn at the end of Task 1 will depend on your health insurance decisions, whether or not you get sick, and chance. At the end of Task 1, we will average your earnings across Year 1 and Year 2 for payment and pay you in real dollars.

We will now begin the first year’s scenario and we will call this Year 1. Before you make your health insurance choice, we would like to give you some additional information. When shopping for insurance in Year 1, please be advised that you have 1 in 3 chance (or 33%) that you will be sick this year. The shaded portion of the pie chart below shows you what a 1 in 3 looks like.


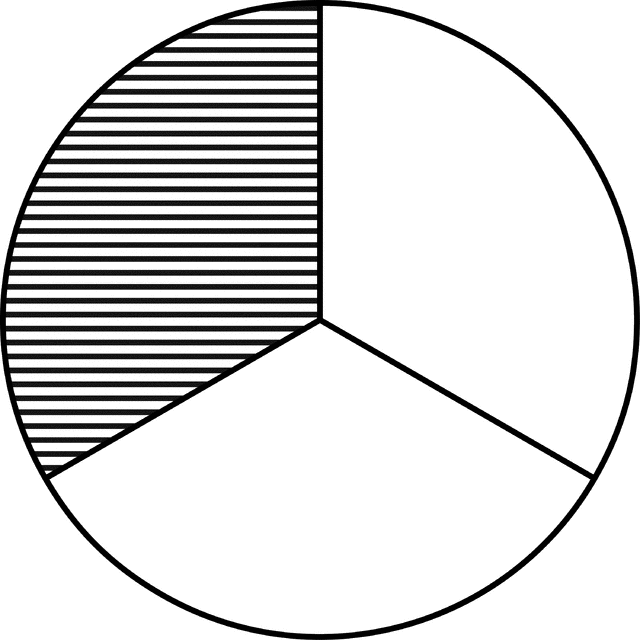


We will randomly determine whether or not you get sick in this scenario *after* you choose your health insurance plan. For example, after you make your health insurance decision, we will roll a fair three-sided die where rolling a “1” means you get sick and rolling a “2” or “3” means you do not get sick.

If you get sick, you will need to see a primary care doctor four times during Year 1. Visiting a primary care doctor cost $500 each time for a total of $2,000. This $2,000 is the only cost you will have if you get sick.

If you do not get sick, you will only have to pay the annual premium if you choose to buy insurance or the tax penalty if you choose not to buy insurance. If you purchase insurance, you will have to pay the annual premium, and, if you get sick, some of the costs of seeing the doctor will be covered by the insurance plan depending on the plan you choose.

If you buy an insurance plan, you have six insurance options (A, B, C, D, E, or F). You also have the option of not buying insurance. However, if you choose not to buy insurance, you will have to pay a tax penalty and, if you get sick, pay the full costs of visiting a primary care doctor. All insurance plans are for individual coverage. That is, imagine you are only buying insurance for yourself. Please make your decision now.

| Plan name | A | B | C | D | E | F | No insurance |
| --- | --- | --- | --- | --- | --- | --- | --- |
| Annual premium or tax penalty | $108 | $156 | $400 | $492 | $1,148 | $1,348 | $695 |
| Annual deductible | $5,500 | $4,500 | $2,250 | $3,350 | $2,000 | $2,000 | Not applicable |
| Out of pocket maximum for the year | $6,350 | $6,350 | $6,350 | $5,500 | $3,000 | $3,000 | Not applicable |
| Cost of 4 doctors’ visits | $2,000 | $2,000 | $2,000 | $2,000 | $2,000 | $2,000 | $2,000 |
| Total you pay if you get sick and need to see the doctor *including* annual premiums or penalties | $2,108 | $2,156 | $2,400 | $2,492 | $3,148 | $3,348 | $2,695 |
| Total you pay if you don’t get sick and don’t need to see the doctor | $108 | $156 | $400 | $492 | $1,148 | $1,348 | $695 |

Please select which health insurance plan you would like to buy for this year or whether you do not wish to buy insurance.

____Plan A

____Plan B

____Plan C

____Plan D

____Plan E

____Plan F

____I don’t want to buy health insurance this year

**Year 2 Prompt (if ill in Year 1)^[[1]](#footnote-1)^**

A year has gone by. It is now Year 2 and you have received another $10,000 Monopoly dollars. Every year there is an open enrollment period, a time when you can shop around for a new insurance plan. If you shop again for health insurance you could save some money but it will take some of your time to shop around.

Before you make your decision, please review the following information. Since you were sick in Year1, you now have an even greater chance of being sick in Year 2. Your chance of being sick in Year 2 is now 4 in 5 (or 80%). See the shaded portion of the pie chart, that’s 4 in 5.


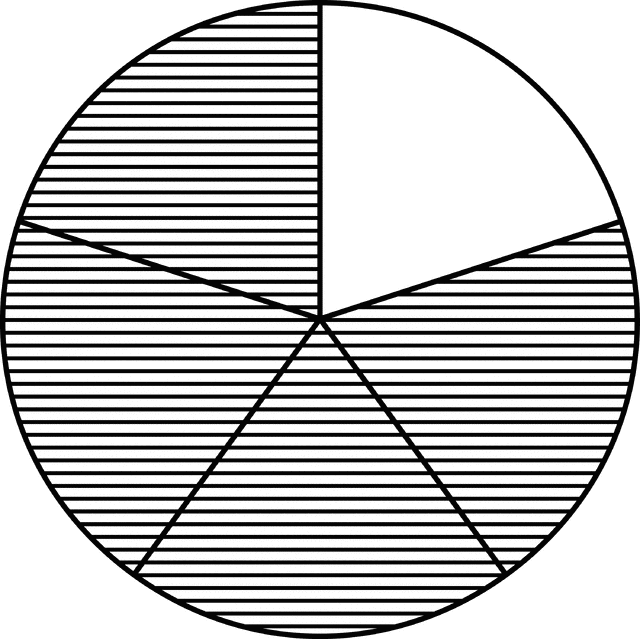


We will randomly determine whether or not you get sick in this scenario *after* you choose your health insurance plan. For example, after you make your health insurance decision, we will roll a fair five-sided die where rolling a “1”,“2”, “3”, or “4” means you get sick and rolling a “5” means you do not get sick.

If you get sick, you will have to go to the emergency room (ER) and then be admitted into the hospital for 15 nights. The bill without insurance will be $5,000 for the ER and $15,000 for the 15 night hospital stay for a total of $20,000. This $20,000 is the only cost you will have if you get sick.

If you do not get sick, you will only have to pay the annual premium if you choose to buy insurance or the tax penalty if you choose not to buy insurance. If you purchase insurance, you will have to pay the annual premium and, if you get sick, some of the costs of going to the ER and being hospitalized will be covered by the insurance plan.

Before making your choice, we wanted to give you the following information:

*{Randomly assigned to one of the following conditions}*

90% of people with your risk of illness choose Plan E

or

Your doctor recommends that people with your risk of illness choose Plan E

or

The government recommends that people with your risk of illness choose Plan E

Which of the following would you like to do?

___ I want to choose the plan recommended for people with my risk of illness by {my

people like me, my doctor, the government}

___ I want to stick with my choice from last year {auto fill plan choice from last year}

___ I want to shop again for health insurance

For those who decide to shop again for health insurance:

You now have the chance to shop again for health insurance. If you buy an insurance plan, you have six insurance options (A, B, C, D, E, or F). You also have the option of not buying insurance. However, if you choose not to buy insurance, you will have to pay a tax penalty and, if you get sick, pay the full costs of hospitalization. All insurance plans are for individual coverage. That is, imagine you are only buying insurance for yourself. Please make your decision now.

| Plan name | A | B | C | D | E | F | No insurance |
| --- | --- | --- | --- | --- | --- | --- | --- |
| Annual Premium or tax penalty | $113 | $164 | $420 | $517 | $1,205 | $1,415 | $730 |
| Annual deductible | $5,500 | $4,500 | $2,250 | $3,350 | $2,000 | $2,000 | Not applicable |
| Out of pocket maximum for the year | $6,350 | $6,350 | $6,350 | $5,500 | $3,000 | $3,000 | Not applicable |
| Cost of the ER visit | $5,000 | $4,725 | $3,075 | $3,763 | $2,900 | $2,900 | $5,000 |
| Cost of the 15 night stay in hospital | $5,750 | $5,250 | $3,000 | $2,250 | $3,000 | $3,000 | $15,000 |
| Total you pay if you get sick after your ER visit and hospital stay *including* annual premiums or tax penalty | $6,463 | $6,514 | $6,495 | $6,017 | $4,205 | $4,415 | $20,730 |
| Total you pay if you don’t get sick and don’t need to go to the ER or hospital | $113 | $164 | $420 | $517 | $1,205 | $1,415 | $730 |

Please select which health insurance plan you would like to buy for this year or whether you do not wish to buy insurance.

____Plan A

____Plan B

____Plan C

____Plan D

____Plan E

____Plan F

____I don’t want to buy health insurance this year

1. If healthy in Year 1, the Year 2 prompt is similar to the Year 1 prompt. [↑](#footnote-ref-1)
